# A Criterion for Distinguishing Temporally Different Dynamical Systems

**DOI:** 10.3390/e27090916

**Published:** 2025-08-29

**Authors:** Evgeny Kagan

**Affiliations:** Department of Industrial Engineering, Faculty of Engineering, Ariel University, Kiryat Ha-Mada, Ariel 4070000, Israel; evganyk@ariel.ac.il

**Keywords:** dynamical systems, internal time, entropy

## Abstract

The paper presents a method for distinguishing dynamical systems with respect to their behavior. The suggested criterion is interpreted as internal time of the ergodic dynamical system, which is a time generated by the system and differs from the external global or reference time. The paper includes a formal definition of the internal time of dynamical system in the form of the entropy ratio, considers its basic properties and gives examples of analysis of dynamical systems.

## 1. Introduction

Analysis of multiagent systems starts with distinguishing the system’s elements—the subsystems which activities differ from the activity of the system and from the other subsystems [1].

Formal criteria for such distinguishing are based on different structural and functional characteristics of the system and depend on the aim of the analysis.

One such criteria that represents dynamics of the system in general is entropy of the system [2,3,4]. As an invariant of the system, entropy is used for distinguishing the system from other systems. The other criterion recently applied for distinguishing the leading agents in the group [5] is based on the structure of the agents’ connections.

The third type of such criteria is local or internal time of the dynamical system; an overview and informal description of internal time is presented in paper [6].

Formal definitions of internal time follow three different approaches (detailed consideration of these approaches is given in Appendix A).

In the approach tracked back to Lévy [7,8], local time is defined as a period during which the states of the system stay in a certain set. The period is measured using the external time (also known as global, universal or reference time) in which the process evolves [9].

The second approach, developed by Prigogine and his group [10,11], considers internal time (or age) as an operator acting on the states of the system in parallel to the operator of the system’s evolution. This approach was developed in several directions; see, e.g., [12] and the references therein.

Finally, in the third approach, developed by Valleé [13,14,15], internal time of a dynamical system is defined as a measure of divergence of the system’s trajectories. This definition is closely related to the Lyapunov criterion of stability of the system’s dynamics [16]. For later development of this approach, see, e.g., [17].

Despite the differences, the indicated formulations have one common disadvantage, which is the need for an external reference time or external indices. In the Lévy approach, external time is used as a measure of the periods; in the Prigogine approach, it is a part of the system’s evolution; and in the Valleé approach, it is hidden in the term “trajectory”.

In the paper, we suggest a new definition of the internal time of an ergodic dynamical system that does not require the external reference time and uses this internal time for distinguishing between temporally different systems.

## 2. Materials and Methods

Let $S=\left(X,\varphi ,\mu \right)$ be an ergodic dynamical system, where X is a differentiable compact manifold called a state space; φ:X→X is an automorphism of X; $\mu :X\to \left[0,1\right]$ is a probability measure; and for any A⊂X, $\mu \left(A\right)=\mu \left(\varphi^{-1}A\right)$ holds.

Let $\alpha =\left\{A_{1},A_{2},\ldots ,A_{n\left(\alpha \right)}\right\}$, $A_{i}\cap A_{j}=\emptyset$ for i≠j, $i,j=1,2,\ldots ,n\left(\alpha \right)$, ${⋃}_{i=1}^{n\left(\alpha \right)}A_{i}=X$, be a finite partition of the space X.

The entropy of partition α with respect to the measure μ is a value [18,19]

$$H\left(\alpha \right)=-\sum_{i=1}^{n\left(\alpha \right)}\mu \left(A_{i}\right)\log\mu \left(A_{i}\right),$$

where log is taken as base 2, and it is assumed that 0log0=0.

Let $\zeta =\left\{Y_{1},Y_{2},\ldots ,Y_{n\left(\zeta \right)}\right\}$ and $\xi =\left\{Z_{1},Z_{2},\ldots ,Z_{n\left(\xi \right)}\right\}$ be two partitions of X. The product of the partitions ζ and ξ is the partition

$$\zeta \vee \xi =\left\{Y_{i}\cap Z_{j}|Y_{i}\in \zeta ,\;Z_{j}\in \xi ,\;i=1,2,\ldots ,n\left(\zeta \right),\;j=1,2,\ldots ,n\left(\xi \right)\right\}.$$

Denote by

$$\varphi^{\nu }\left(\alpha \right)=\left\{\varphi^{\nu }\left(A_{1}\right),\varphi^{\nu }\left(A_{2}\right),\ldots ,\varphi^{\nu }\left(A_{n\left(\alpha \right)}\right)\right\},\;\nu =0,\;1,\;2,\ldots ,$$

the νth application of the automorphism φ to partition α, where we assume that $\varphi^{0}\left(\alpha \right)=\alpha$, and by

$$\alpha_{N}\left(\varphi \right)={⋁}_{v=0}^{N}\varphi^{v}\left(\alpha \right).$$

a partition obtained by the multiplication of N+1 partitions $\varphi^{0}\left(\alpha \right),\;\varphi^{1}\left(\alpha \right),\ldots ,\varphi^{N}\left(\alpha \right)$ obtained by iterative applications of the automorphism φ to the partition α.

Denote by

$$H\left[\alpha_{N}\left(\varphi \right)\right]=H\left[{⋁}_{v=0}^{N}\varphi^{v}\left(\alpha \right)\right]=H\left[\varphi^{N}\left(\alpha \right)\;|\;{⋁}_{v=0}^{N-1}\varphi^{v}\left(\alpha \right)\right]$$

an entropy of the partition $\alpha_{N}\left(\varphi \right)$.

**Definition** **1** ([18,19,20])**.** *The limiting value*

$$h\left(\varphi ,\alpha \right)=\underset{N\to \infty }{\lim}\frac{1}{N}H\left[\alpha_{N}\left(\varphi \right)\right],$$

*is called the entropy of the system* S *with respect to time, and its supremum*

$$h\left(\varphi \right)=\sup_{\alpha }h\left(\varphi ,\alpha \right),$$

*taken over all finite measurable partitions of* X *is called the entropy of dynamical system* S*.*

Originally, the concept of entropy of a dynamical system was suggested by Kolmogorov [2,3] and formulated in terms of the phase flow $\left\{g^{t}\right\}$, where $g^{t}:X\to X$ is a trajectory of the system in X at time t. Later, Sinai [4] suggested the formulation used above. For a detailed consideration of the theory of entropy in the framework of dynamical systems, see books [18,19,20]; a brief overview of these entropies is given in Appendix B.

Also, below we will need the following facts.

Let $\zeta =\left\{Y_{1},Y_{2},\ldots ,Y_{n\left(\zeta \right)}\right\}$ and $\xi =\left\{Z_{1},Z_{2},\ldots ,Z_{n\left(\xi \right)}\right\}$ be two partitions of X. If each set Z∈ξ is a subset of some set Y∈ζ, then it is said that the partition ξ is a refinement of the partition ζ, which is written as ξ≽ζ.

**Lemma** **1** ([19])**.** *If* ξ≽ζ*, then* $H\left(\zeta \right)\leq H\left(\xi \right)$.

Let ζ∨ξ be a multiplication of the partitions ζ and ξ. Since each set W∈ζ∨ξ is a subset of some set Y∈ζ and of some set Z∈ξ, partition ζ∨ξ is a refinement of both ζ and ξ, that is, ζ∨ξ≽ζ and ζ∨ξ≽ξ.

Then, following Lemma 1, $H\left(\zeta \right)\leq H\left(\zeta \vee \xi \right)$ and $H\left(\xi \right)\leq H\left(\zeta \vee \xi \right)$.

## 3. Results

Internal time of a dynamical system is defined as follows. Let α be a partition that provides the supremum of the entropy $h\left(\varphi ,\alpha \right)$, that is,

$$h\left(\varphi ,\alpha \right)=h\left(\varphi \right).$$

**Definition** **2.** *The value*

$$\tau_{N}\left(\varphi \right)=\frac{1}{H\left[\varphi^{0}\left(\alpha \right)\right]}\left(H\left[\varphi^{N}\left(\alpha \right)\vee \varphi^{N-1}\left(\alpha \right)\right]-H\left[\varphi^{N-1}\left(\alpha \right)\right]\right),$$

*is called the internal time of dynamical system S at iteration N, and the limit*

$$\tau \left(\varphi \right)=\underset{N\to \infty }{\lim}\tau_{N}\left(\varphi \right).$$

 *is called the internal time of dynamical system* S*.*

The suggested definition follows a widely accepted understanding of time as some form of the change in entropy. The idea of such definitions is to provide a quantitative parameter that represents the system’s stability and periodicity. For example, an already mentioned Lyapunov criterion [16] represents the divergence of the system’s trajectories but does not relate it with the entropy of the system, which is one of the main criteria for distinguishing the systems [19,20]. The suggested definition attempts to bridge this gap.

To clarify the introduced definition, let us consider several examples.

**Example** **1** (circle rotations)**.** *Assume that in the system* S*, the set* X *is a circle of unit radius and the subsets* A⊂X *are the arcs of the circle* X*. The measure* $\mu \left(A\right)$ *of arc* A *is defined as a length of* A *divided by* 2π*, and the automorphism* φ *defines rotations* 

$$\varphi \left(x,\theta \right)=\left(x+\theta \right)\mathrm{mod}2\pi ,\;x\in X,$$

*of the circle* X *to the angle* θ*.*

*The entropy of such a system is* [18,20]

$$h\left(\varphi \right)=0,$$

*and we can choose any partition* $\alpha =\varphi^{0}\left(\alpha ,\theta \right)$.

*Let* $\varphi^{0}\left(\alpha ,\theta \right)=\left\{A_{1},A_{2}\right\}$ *be a partition of* X *such that* $A_{1}$ *is a left semicircle and* $A_{2}$ *is a right semicircle and let* φ *be a rotation of* X *to the angle* $\theta =\frac{\pi }{2}$*. Then,*

$$\varphi^{1}\left(\alpha ,\theta \right)=\left\{\varphi^{1}\left(A_{1},\theta \right),\varphi^{1}\left(A_{2},\theta \right)\right\}$$

*is a partition such that* $\varphi^{1}\left(A_{1},\theta \right)$ *is a bottom semicircle and* $\varphi^{1}\left(A_{2},\theta \right)$ *is a top semicircle.*

*Hence,* $\varphi^{1}\left(\alpha ,\theta \right)\vee \varphi^{0}\left(\alpha ,\theta \right)$ *is a partition of* X *into four equivalent disjoint arcs.*

*The entropy of the partitions* $\varphi^{0}\left(\alpha ,\theta \right)$ *and* $\varphi^{1}\left(\alpha ,\theta \right)$ *is* 

$$H\left[\varphi^{0}\left(\alpha ,\theta \right)\right]=H\left[\varphi^{1}\left(\alpha ,\theta \right)\right]=-\frac{1}{2}\log\frac{1}{2}-\frac{1}{2}\log\frac{1}{2}=1,$$

*and the entropy of the partition* $\varphi^{1}\left(\alpha ,\theta \right)\vee \varphi^{0}\left(\alpha ,\theta \right)$ *is* 

$$H\left(\varphi^{1}\left(\alpha ,\theta \right)\vee \varphi^{0}\left(\alpha ,\theta \right)\right)=-\frac{1}{4}\log\frac{1}{4}-\frac{1}{4}\log\frac{1}{4}-\frac{1}{4}\log\frac{1}{4}-\frac{1}{4}\log\frac{1}{4}=2.$$

*Consequently, the internal time of this system at the first iteration* N=1 *is* 

$$\tau_{1}\left(\varphi \right)=2-1=1.$$

*At the next step* N=2*, in the partition*

$$\varphi^{2}\left(\alpha ,\theta \right)=\left\{A_{1},A_{2}\right\}$$

$A_{1}$ *is a right semicircle and* $A_{2}$ *is a left semicircle, and, similar to above, partition* $\varphi^{2}\left(\alpha ,\theta \right)\vee \varphi^{1}\left(\alpha ,\theta \right)$ *includes four equivalent disjoint arcs.*

*Thus, the entropies are* 

$$H\left[\varphi^{1}\left(\alpha ,\theta \right)\right]=1\;\;\;\mathrm{and}\;\;\;H\left(\varphi^{2}\left(\alpha ,\theta \right)\vee \varphi^{1}\left(\alpha ,\theta \right)\right)=2,$$

*and the internal time at the step* N=2 *is* 

$$\tau_{2}\left(\varphi \right)=1.$$

*The same partitions and the values of entropies and distances are obtained for any* N=1, 2,… *Thus, the internal time of the system is* 

$$\tau \left(\varphi \right)=1.$$

*Note that the internal time of this system depends on the angle* θ.

**Example** **2.** *In the system* S*, let the set* $X=\left(\left.0,\;1\right]\right.$ *be a unit interval without zero and let measure* μ *be a length of the subintervals of* X*. The automorphism* φ *is defined as* 

$$\varphi \left(x,m\right)=\frac{1}{m}x,\;x\in X.$$

*Let* 

$$\alpha =\left\{\left(\left.\frac{j-1}{m},\frac{j}{m}\right]\right.\;|\;j=1,\ldots ,\;m\right\}$$

*be a partition of* X*. Then,*

$$\alpha_{N}\left(\varphi \right)=\left\{\left(\left.\frac{j-1}{m^{N}},\frac{j}{m^{N}}\right]\right.\;|\;j=1,\;2,\;\ldots ,\;m^{N}\right\},$$

*the entropy of* $\alpha_{N}\left(\varphi \right)$ *is* 

$$H\left[\alpha_{N}\left(\varphi \right)\right]=-\sum_{j=1}^{m^{N}}\frac{1}{m^{N}}\log\frac{1}{m^{N}}=N\log m$$

*and of the system is* 

$$h\left(\varphi \right)=\sup_{\alpha }\underset{N\to \infty }{\lim}\frac{1}{N}H\left[\alpha_{N}\left(\varphi \right)\right]=\log m.$$

*Let* m=2*. Then,*

$$\varphi^{0}\left(\alpha ,2\right)=\alpha =\left\{\left(\left.0,\frac{1}{2}\right]\right.,\;\left(\left.\frac{1}{2},1\right]\right.\right\},\;\varphi^{1}\left(\alpha ,2\right)=\left\{\left(\left.0,\frac{1}{4}\right]\right.,\;\left(\left.\frac{1}{4},\frac{1}{2}\right]\right.,\left(\left.\frac{1}{2},\frac{3}{4}\right]\right.,\;\left(\left.\frac{3}{4},1\right]\right.\right\},\;\varphi^{2}\left(\alpha ,2\right)=\left\{\left(\left.0,\frac{1}{8}\right]\right.,\;\left(\left.\frac{1}{8},\frac{1}{4}\right]\right.,\left(\left.\frac{1}{4},\frac{3}{8}\right]\right.,\;\left(\left.\frac{3}{8},\frac{1}{2}\right]\right.,\left(\left.\frac{1}{2},\frac{5}{8}\right]\right.,\;\left(\left.\frac{5}{8},\frac{3}{4}\right]\right.,\left(\left.\frac{3}{4},\frac{7}{8}\right]\right.,\;\left(\left.\frac{7}{8},1\right]\right.\right\}.$$

*The entropies of these partitions are* 

$$H\left[\varphi^{0}\left(\alpha ,2\right)\right]=-2\left(\frac{1}{2}\log\frac{1}{2}\right)=1,\;H\left[\varphi^{1}\left(\alpha ,2\right)\right]=-4\left(\frac{1}{4}\log\frac{1}{4}\right)=2,\;H\left[\varphi^{2}\left(\alpha ,2\right)\right]=-8\left(\frac{1}{8}\log\frac{1}{8}\right)=3.$$

*Thus, the internal time at the first iteration is* 

$$\tau_{1}\left(\varphi \right)=2-1=1\;\;\;\mathrm{and}\;\;\;\tau_{2}\left(\varphi \right)=3-2=1.$$

*The same calculations for the next iterations show that* 

$$\tau_{N}\left(\varphi \right)=1,$$

*and the internal time of the system is* 

$$\tau \left(\varphi \right)=1.$$

*For detailed calculations, see Appendix C*.

*Note that despite an infinite increase of the entropy of the system, its internal time is finite and is equivalent to the internal time of the rotations of the circle.*

**Example** **3** (Bernoulli shift)**.** *Let* $X=\left[0,\;1\right]$ *be a union of two open intervals. Bernoulli shift* φ *on* X *is defined by the iterative formula* 

$$x_{N+1}=2x_{N}\mathrm{mod}1=\left\{ \begin{array}{ll} 2x_{N} & \mathrm{if}\;x_{N}\in \left[0,\frac{1}{2}\right)\\ 2x_{N}-1 & \mathrm{if}\;x_{N}\in \left[\frac{1}{2},1\right] \end{array}.\right.$$

*Let* 

$$\alpha =\left\{A_{j}\;|\;j=1,\ldots ,\;m\right\}$$

*be a partition of* X*. The measure* μ *is defined as a probability that the state* $x_{N}\in A_{j}$.

*Let* m=2 *and assume that* 

$$\varphi^{0}\left(\alpha \right)=\alpha =\left\{\left[\left.0,\frac{1}{2}\right)\right.,\;\left[\frac{1}{2},1\right]\right\}.$$

*Then,* 

$$\varphi^{1}\left(\alpha \right)=\left\{\left[\left.0,\frac{1}{2}\right)\right.,\;\left[\frac{1}{2},1\right]\right\},\;\;\;\varphi^{2}\left(\alpha \right)=\left\{\left[\left.0,\frac{1}{2}\right)\right.,\;\left[\frac{1}{2},1\right]\right\},$$

*and so on.*

*The entropies of these partitions are* 

$$H\left[\varphi^{N}\left(\alpha \right)\right]=-2\left(\frac{1}{2}\log\frac{1}{2}\right)=1,$$

*Thus, the internal time at the* N*th iteration is* 

$$\tau_{N}\left(\varphi \right)=1-1=0$$

*and the internal time of the system is also* 

$$\tau \left(\varphi \right)=0$$

*For detailed calculations, see Appendix C.*

*The same holds true for any partition* α *to* m *subsets such that* $\mu \left(A_{j}\right)=\mu \left(A_{k}\right)$ j,k=1,2,…,m*.*

*Now assume that* 

$$\varphi^{0}\left(\alpha \right)=\alpha =\left\{\left[\left.0,\frac{1}{3}\right)\right.,\;\left[\frac{1}{3},1\right]\right\}.$$

*Then,* 

$$\varphi^{1}\left(\alpha \right)=\left\{\left[\left.0,\frac{2}{3}\right)\right.,\;\left[\frac{2}{3},1\right]\right\},\;\varphi^{2}\left(\alpha \right)=\left\{\left[\left.0,\frac{1}{3}\right)\right.,\;\left[\frac{1}{3},1\right]\right\},$$

*and so on.*

*The entropies of these partitions are* 

$$H\left[\varphi^{N}\left(\alpha \right)\right]=\log3-\frac{2}{3},\;N=0,\;1,\;2,\ldots$$

*The multiplications of these partitions are* 

$$\varphi^{N}\left(\alpha \right)\vee \varphi^{N-1}\left(\alpha \right)=\left\{\left[\left.0,\frac{1}{3}\right)\right.,\left[\left.\frac{1}{3},\frac{2}{3}\right)\right.,\;\left[\frac{2}{3},1\right]\right\},$$

*and the entropies of the multiplications are* 

$$H\left[\varphi^{N}\left(\alpha \right)\vee \varphi^{N-1}\left(\alpha \right)\right]=\log3,\;N=0,\;1,\;2,\ldots$$

*Thus, internal time at the* N*th iteration is* 

$$\tau_{N}\left(\varphi \right)=\frac{1}{\log3-\frac{2}{3}}\left(\log3-\left(\log3-\frac{2}{3}\right)\right)=\frac{2}{3\log3-2}\approx 0.726$$

*and the internal time of the system is* 

$$\tau \left(\varphi \right)=\frac{2}{3\log3-2}\approx 0.726.$$

*For the other partitions, internal times are calculated similarly.*

It is easy to demonstrate that the internal time has the following properties.

**Theorem** **1.** $\tau_{N}\left(\varphi \right)\geq 0$ *for any N=1,2,…*

**Proof.**  By the properties of entropy,

$$H\left[\varphi^{0}\left(\alpha \right)\right]\geq 0,$$

and since α includes at least two non-empty sets with positive measures:

$$H\left[\varphi^{0}\left(\alpha \right)\right]\neq 0.$$

Since

$$\varphi^{N}\left(\alpha \right)\vee \varphi^{N-1}\left(\alpha \right)≽\varphi^{N-1}\left(\alpha \right)$$

by Lemma 1, the following holds:

$$H\left[\varphi^{N}\left(\alpha \right)\vee \varphi^{N-1}\left(\alpha \right)\right]\geq H\left[\varphi^{N-1}\left(\alpha \right)\right].$$

Hence,

$$H\left[\varphi^{N}\left(\alpha \right)\vee \varphi^{N-1}\left(\alpha \right)\right]-H\left[\varphi^{N-1}\left(\alpha \right)\right]\geq 0$$

and the internal time is non-negative. □

The next theorem defines a bound of applicability of internal time in the analysis of the systems.

**Theorem** **2.** *If system* $S=\left(X,\varphi ,\mu \right)$ *is periodic, then $\tau_{N}\left(\varphi \right)<\infty$*.

**Proof.**  In the periodic system for some k>0, the following holds:

$$\varphi^{N+k}\left(\alpha \right)=\varphi^{N}\left(\alpha \right),\;N=0,\;1,\;2,\ldots$$

Hence, $H\left[\varphi^{N}\left(\alpha \right)\vee \varphi^{N-1}\left(\alpha \right)\right]-H\left[\varphi^{N-1}\left(\alpha \right)\right]$ is periodic with period k. □

Along with this, note that applicability of the internal time is not limited by periodic systems and can be used for any system with distortion which trajectories return to an ε-surrounding of some point in X.

Let $S_{1}=\left(X_{1},\varphi_{1},\mu_{1}\right)$ and $S_{2}=\left(X_{2},\varphi_{2},\mu_{2}\right)$ be two dynamical systems.

**Definition** **3.** *We say that the systems* $S_{1}\;and\;S_{2}$*are temporally equivalent if for each N=0, 1, 2,…, either*

$$\tau_{N}\left(\varphi_{1}\right)=\tau_{N}\left(\varphi_{2}\right)=0$$

*or the ratios*

$$\gamma_{N}\left(\varphi_{1},\varphi_{2}\right)=\frac{\tau_{N}\left(\varphi_{1}\right)}{\tau_{N}\left(\varphi_{2}\right)},\;N=0,\;1,\;2,\ldots$$

*are rational numbers.*

Informally speaking, the suggested criterion means that the clocks based on the behaviourally equivalent systems can be used together and their results can be compared with any precision. In contrast, results of the clocks based on behaviourally different systems can be compared with limited precision only.

Note that in practice, recognition of rational or irrational ratios is possible only for simple cases, while in general comparison of internal times can be conducted with certain finite precision. Then, an additional check of convergence of the sequences $\tau_{N}\left(\varphi_{1}\right)$ and $\tau_{N}\left(\varphi_{2}\right)$, N=0, 1, 2, ..., is required.

The sequences $\tau_{N}\left(\varphi_{1}\right)$ and $\tau_{N}\left(\varphi_{2}\right)$, N=0, 1, 2, ..., of internal times can also be compared by appropriate statistical methods. However, since the elements of each sequence are not independent, such a comparison is strongly limited.

**Theorem** **3.** *If the systems* $S_{1}\;\mathrm{and}\;S_{2}$ *are isomorphic, then they are temporally equivalent.*

In other words, behavioral equivalence is weaker than an isomorphism of the systems.

**Proof.**  Let $u:X_{1}\to X_{2}$ be an isomorphism of the systems $S_{1}$ and $S_{2}$, which means that $\varphi_{2}=u\varphi_{1}u^{-1}$. Thus, for each N=0, 1, 2,…,

$$\varphi_{2}^{N}\left(u\alpha \right)=u\varphi_{1}^{N}u^{-1}\left(u\alpha \right)=u\varphi_{1}^{N}\left(\alpha \right).$$

Since u is an isomorphism, it gives (N=1, 2,…)

$$H\left[u\varphi_{1}^{N-1}\left(\alpha \right)\right]=H\left[\varphi_{1}^{N-1}\left(\alpha \right)\right],\;H\left[u\varphi_{1}^{N}\left(\alpha \right)\vee u\varphi_{1}^{N-1}\left(\alpha \right)\right]=H\left[\varphi_{1}^{N}\left(\alpha \right)\vee \varphi_{1}^{N-1}\left(\alpha \right)\right].$$

Hence, for each N=1, 2,…, the following holds:

$$\begin{array}{cc} \tau_{N}\left(\varphi_{2}\right)\; & =\frac{1}{H\left[\;\varphi_{2}^{0}\left(u\alpha \right)\right]}\left(H\left[\varphi_{2}^{N}\left(u\alpha \right)\vee \varphi_{2}^{N-1}\left(u\alpha \right)\right]-H\left[\;\varphi_{2}^{N-1}\left(u\alpha \right)\right]\right)\\ & =\frac{1}{H\left[u\varphi_{1}^{0}\left(\alpha \right)\right]}\left(H\left[u\varphi_{1}^{N}\left(\alpha \right)\vee u\varphi_{1}^{N-1}\left(\alpha \right)\right]-H\left[u\varphi_{1}^{N-1}\left(\alpha \right)\right]\right)\\ & =\frac{1}{H\left[\varphi_{1}^{0}\left(\alpha \right)\right]}H\left[\varphi_{1}^{N}\left(\alpha \right)\vee \varphi_{1}^{N-1}\left(\alpha \right)\right]-H\left[\varphi_{1}^{N-1}\left(\alpha \right)\right]\\ & =\tau_{N}\left(\varphi_{1}\right). \end{array}$$

which is required. □

Let us illustrate the use of the suggested criteria.

**Example** **4.** *Consider a pair of Tsetlin automata* $A_{1}$ *and* $A_{2}$ *acting in the stochastic environments:*

$$E_{1}=\left\{p_{11},p_{12},\ldots ,p_{1m}\right\}\;\mathrm{and}\;E_{2}=\left\{p_{21},p_{22},\ldots ,p_{2m}\right\}.$$

*where* $p_{k1}+p_{k2}+\ldots +p_{km}=1$*,* k=1, 2.

*The activity of each automaton* $A_{k}$ *is defined as follows* [21]*. Assume that the states space of automaton* $A_{k}$ *is* 

$$S_{k}=\left\{s_{k1},\;s_{k2},\ldots ,s_{kn}\right\}$$

*and assume that in each state* $s_{ki}$*, the automaton can conduct one of the actions* $a_{kj}$ *from the set* 

$$A_{k}=\left\{a_{k1},\;a_{k2},\ldots ,a_{km}\right\}$$

*If automaton* $A_{k}$ *conducts an action* $a_{kj}$*, then with probability* $p_{kj}$*, its outcome is* $o_{kj}=1$ *(payoff), and with probability* $q_{kj}=1-p_{kj}$*, its outcome is* $o_{kj}=0$ *(reward), where probabilities* $p_{kj}$*,* j=1,2,…,m*, are defined by the environment* $E_{k}$, k=1, 2.

*Transitions of automaton* $A_{k}$ *are defined by two matrices:*

$$B^{k}\left(1\right)=\left\|b_{ij}^{k}\left(1\right)\right\|\;\;\;\mathrm{and}\;\;\;B^{k}\left(0\right)=\left\|b_{ij}^{k}\left(0\right)\right\|$$

*such that each row of the matrix* $b^{k}\left(1\right)$ *includes a single element* $b_{ij}^{k}\left(1\right)=1$ *and each row of the matrix* $b^{k}\left(0\right)$ *includes a single element* $b_{ij}^{k}\left(0\right)=1$*; all other elements of the matrices are zeros.*

*Thus, being in the state* $s_{ki}$ *and receiving the outcome* $o_{kj}=1$*, the automaton transitions to the state* $s_{kj}$ *prescribed by the element* $b_{ij}^{k}\left(1\right)=1$*, and while receiving the outcome* $o_{kj}=0$*, the automaton transitions to the state* $s_{kj}$ *prescribed by the element* $b_{ij}^{k}\left(0\right)=1$.

*Assume that automaton* $A_{k}$ *is in the state* $s_{ki}$*. Then, probability* $\rho_{ij}^{k}$ *of transition from the state* $s_{ki}$ *to the state* $x_{kj}$ *is* 

$$\rho_{ij}^{k}=p_{kl}b_{ij}^{k}\left(1\right)+q_{kl}b_{ij}^{k}\left(0\right),\;k\;=1,\;2,\;l=1,\;2,\;\ldots ,\;m,\;i,j=1,\;2,\;\ldots ,\;n.$$

*Let each automaton* $A_{k}$ *be a binary automaton with* $S_{k}=\left\{0,\;1\right\}$ *acting in the environment with two states* $E_{k}=\left\{p_{k1},p_{k2}\right\}$, k=1, 2.

*Transitions of the automata are defined by the matrices* 

$$b^{k}\left(1\right)=\left(\begin{array}{cc} 0 & 1\\ 1 & 0 \end{array}\right)\;\;\;\mathrm{and}\;\;\;b^{k}\left(0\right)=\left(\begin{array}{cc} 1 & 0\\ 0 & 1 \end{array}\right),$$

*which specify that if automaton* $A_{k}$ *receives outcome* $o_{k}=1$*, then it changes its state to the opposite and if automaton* $A_{k}$ *receives outcome* $o_{k}=0$*, then it stays in its current state.*

*In terms of dynamical systems, such activity is defined by the* xor *function:* 

$$s_{k}^{N}=\mathrm{xor}\left(s_{k}^{N-1},o_{k}^{N-1}\right),\;N=1,\;2,\;\ldots$$

*Matrices of transition probabilities are* 

$$\rho^{k}=\left(\begin{array}{cc} \rho_{11}^{k} & \rho_{12}^{k}\\ \rho_{21}^{k} & \rho_{22}^{k} \end{array}\right)=\left(\begin{array}{cc} q_{k1} & p_{k1}\\ p_{k2} & q_{k2} \end{array}\right).$$

*The steady state probabilities of automaton* $A_{k}$ *are* 

$$r_{k1}=\frac{p_{k2}}{p_{k1}+p_{k2}}\;\;\;\mathrm{and}\;\;\;r_{k2}=\frac{p_{k1}}{p_{k1}+p_{k2}},$$

*and the expected outcome of the automaton* $A_{k}$ *is* 

$$E\left(o_{k}\right)=\frac{2p_{k1}p_{k2}}{p_{k1}+p_{k2}},\;k\;=1,\;2.$$

*Each automaton* $A_{k}$, k=1, 2*, is associated with the dynamical system* $S_{k}=\left(X_{k},\varphi_{k},\mu_{k}\right)$*, where* $X_{k}=\left[0,\;1\right]$*, automorphism* $\varphi_{k}:X_{k}\to X_{k}$ *is defined by the matrices* $B^{k}\left(0\right)$ *and* $B^{k}\left(1\right)$*, and measure* $\mu_{k}$ *coincides with the steady state probabilities* $r_{k}$.

*Let* $\alpha_{k}=\left\{A_{k1},A_{k2}\right\}$ *be a partition of the space* $X_{k}$ *such that* $\mu_{k}\left(A_{k1}\right)=r_{k1}$ *and* $\mu_{k}\left(A_{k2}\right)=r_{k2}$.

*Then, internal time* $\tau_{N}^{k}=\tau_{N}\left(\varphi_{k}\right)$ *per iteration* N=0, 1, 2, … *and internal time* $\tau^{k}=\tau \left(\varphi_{k}\right)$ *of the system* $S_{k}$ *define the corresponding internal times of the Tsetlin automaton* $A_{k}$.

*If the automata* $A_{1}$ *and* $A_{2}$ *act in the equivalent environments* $E_{1}=E_{2}$*, then from Definition 3, it follows that they are temporally equivalent.*

*In fact, if* $E_{1}=E_{2}$*, then*

$$r_{11}=r_{21}\;\;\;\mathrm{and}\;\;\;r_{12}=r_{22}.$$

*Hence, in the partitions* $\alpha_{1}=\left\{A_{11},A_{12}\right\}$ *and* $\alpha_{2}=\left\{A_{21},A_{22}\right\}$:

$$\mu_{1}\left(A_{11}\right)=\mu_{2}\left(A_{21}\right)\;\;\;\mathrm{and}\;\;\;\mu_{1}\left(A_{12}\right)=\mu_{2}\left(A_{22}\right).$$

*Since, by definition,* $\varphi_{1}\equiv \varphi_{2}$*, for each* N=1, 2, …*, the following holds:*

$$H\left[\varphi_{1}^{N}\left(\alpha_{1}\right)\right]=H\left[\varphi_{2}^{N}\left(\alpha_{2}\right)\right]\;\;\;\mathrm{and}\;\;\;H\left[\varphi_{1}^{N}\left(\alpha_{1}\right)\vee \varphi_{1}^{N-1}\left(\alpha_{1}\right)\right]=H\left[\varphi_{2}^{N}\left(\alpha_{2}\right)\vee \varphi_{2}^{N-1}\left(\alpha_{2}\right)\right],$$

*which results in the equivalence of the internal times:*

$$\tau_{N}\left(\varphi_{1}\right)=\tau_{N}\left(\varphi_{2}\right),\;N=0,\;1,\;2,\;\ldots$$

*On the other hand, if the automata act in different environments* $E_{1}\neq E_{2}$*, then the behavioral equivalence of the automata* $A_{1}$ and $A_{2}$ *is defined by the ratio* $\gamma_{N}\left(\varphi_{1},\varphi_{2}\right)$ *of their internal times per iteration. For example, if the environments* $E_{1}$ *and* $E_{2}$ *are such that* 

$$\alpha_{1}=\left\{\left[\left.0,\frac{1}{3}\right)\right.,\;\left[\frac{1}{3},1\right]\right\}\;\;\;\mathrm{and}\;\;\;\alpha_{2}=\left\{\left[\left.0,\frac{2}{3}\right)\right.,\;\left[\frac{2}{3},1\right]\right\},$$

*then the automata* $A_{1}$ *and* $A_{2}$ *are temporally equivalent, but if they are such that* 

$$\alpha_{1}=\left\{\left[\left.0,\frac{1}{3}\right)\right.,\;\left[\frac{1}{3},1\right]\right\}\;\;\;\mathrm{and}\;\;\;\alpha_{2}=\left\{\left[\left.0,\frac{1}{4}\right)\right.,\;\left[\frac{1}{4},1\right]\right\},$$

*then the automata* $A_{1}$ *and* $A_{2}$ *are temporally different.*

Let us demonstrate the behavioral equivalence of a binary Tsetlin automaton and Bernoulli shifts.

**Lemma** **2.** *Let* A *be a Tsetlin automaton acting in the environment $E=\left\{p_{1},p_{2}\right\}$. Then there exist Bernoulli shifts $S_{1}$* *and $S_{2}$* *such that with certain probability, behavior of A* *is equivalent to the behavior of one of the shifts $S_{1}$* *and $S_{2}$*.

**Proof.**  To prove the lemma, we will construct the required Bernoulli shifts $S_{1}$ and $S_{2}$ given the automaton A and its environment E.

Given the environment $E=\left\{p_{1},p_{2}\right\}$, the steady state probabilities of A are

$$r_{1}=\frac{p_{2}}{p_{1}+p_{2}}\;\;\;\mathrm{and}\;\;\;r_{2}=\frac{p_{1}}{p_{1}+p_{2}}.$$

Define the states space $X=\left[0,\;1\right]$ and two partitions

$$\alpha_{1}=\left\{\left[\left.0,r_{1}\right)\right.,\;\left[r_{1},1\right]\right\}\;\;\;\mathrm{and}\;\;\;\alpha_{2}=\left\{\left[\left.0,r_{2}\right)\right.,\;\left[r_{2},1\right]\right\}.$$

Then, the shift $S_{1}$ acting on the partition $\alpha_{1}$ corresponds to the activity of the automaton A while it receives outcome o=1 (payoff) and the shift $S_{2}$ acting on the partition $\alpha_{2}$ corresponds to the activity of the automaton A while it receives outcome o=0 (reward).

Finally, recalling that the probability of receiving outcome o=1 is $r_{1}$ and the probability of receiving outcome o=0 is $r_{2}$, we obtain the statement of the lemma. □

## 4. Discussion

In the paper, we consider internal time of an ergodic dynamical system as an operational criterion for distinguishing subsystems that demonstrate autonomous behavior. A rich collection of the most influential ideas and approaches to understanding time was published by Jaroszkiewicz in the book [22], which continues the earlier seminal philosophical work by Whitrow [23], who, among other ideas, contraposed the concepts “arrow of time” and “circle of time”.

In the natural sciences, the concept of time is usually considered together with the concept of space. The most known popular sources about time written by physicists are probably the book by Hawking [24] and the books by Carroll [25] and by Rovelli [26]. An influential source about the arrow of time is the book by Zeh [27].

The ideas considered in this paper were inspired by the concept of time derived from the steps of logical implications suggested by Reichenbach [28]. Following classification by Jaroszkiewicz [22], such an approach can be considered in the framework of “contextual truth” without referencing “external reality”.

After publication of the books by Haken [29] and Prigogine [30] and further studies of non-linear dynamical systems, it became clear that each dynamical system generates a certain internal time that characterizes and is characterized by the system’s behavior and its interactions with the environment.

From these studies, it also follows that internal time of linear systems is, in a certain sense, proportional to the external global time, and the internal time of non-linear systems can strongly differ from the global time and even can have varying scales.

Appendix A briefly presents the three most influential definitions of internal time that inspired this paper.

As indicated in the Introduction, the need for the criterion for distinguishing the systems with respect to their behavior arose in the analysis of multiagent systems. For example, in the consideration of swarm dynamics, it is required to distinguish the agents that behave differently from the others. Such a task is similar to the task of distinguishing the pacemakers as it appears in the analysis of active media or on the analysis of networks of spiking neurons. In addition, the suggested criterion will probably also be useful in studies of symbolic dynamics; however, this issue requires additional studies.

## 5. Conclusions

In the paper, we suggest a criterion for distinguishing temporally different systems that is required for analysis of groups of autonomous agents.

The suggested criterion is based on the entropy of an ergodic dynamical system and has the meaning of internal time of the system that characterizes dynamics of the system. Then, if two systems have comparable internal times, which means that the ratio of the times is a rational number, then the systems are temporally equivalent, and if this ratio is irrational, then the systems are temporally different.

Calculations of the internal time and uses of the criterion are illustrated by numerical examples.

## Data Availability

No new data were created or analyzed in this study. Data sharing is not applicable to this article.

## Appendix A

### Appendix A.1. Local Time of Brownian Motion

Let $w\left(\tau \right)$, τ≥0, be one-dimensional Brownian motion and let B be a σ-algebra of the Borel sets from R.

Denote by $\mu_{t}\left(A\right)$ a Lebesgue measure of time up to t during which the trajectory of the process $w\left(\tau \right)$ is in the set A∈B. Then,

$$\mu_{t}\left(A\right)=\int_{0}^{t}I_{A}\left(w\left(\tau \right)\right)d\tau ,$$

where $I_{A}\left(w\left(\tau \right)\right)$ is an indicator function of the set A.

Lévy demonstrated [7] that there exists a density function $l_{t}$ such that with probability one for all trajectories of the process $w\left(\tau \right)$, any time t>0 and any set A∈B, it holds true that

$$\mu_{t}\left(A\right)=\int_{A}l_{t}\left(x\right)dx,$$

This density $l_{t}\left(x\right)$ is called the local time of Brownian motion $w\left(\tau \right)$ in the point x up to time t.

Finally, Trotter [31] proved that with probability one, there exists a process $t:\left[\left.0,\infty \right)\times R\to R\right.$ such that for any x∈R,

$$t\left(t,x\right)=l_{t}\left(x\right).$$

The process t is called *local time* of Brownian motion $w\left(\tau \right)$. For the fixed t, the process t is a Markov process on the points x.

A detailed overview of local time t was published by Borodin [9].

### Appendix A.2. Operator of Internal Time

Let $\left(p,q\right)$ be canonical coordinates of the n-dimensional dynamical system, and assume that its evolution is defined by following the system of Hamilton equations:

$${\dot{q}}_{i}=\;\frac{\partial H}{\partial p_{i}},\;{\dot{p}}_{i}=\;-\frac{\partial H}{\partial q_{i}},\;i=\;1,2,\ldots ,n,$$

where ${\dot{q}}_{i}=dq_{i}/dt$; ${\dot{p}}_{i}=dp_{i}/dt$; and for initial time $t\;=t_{0}$, the values $q_{i}\left(t_{0}\right)=q_{i}^{0}$ and $p_{i}\left(t_{0}\right)=p_{i}^{0}$ are specified.

Denote by

$$L=-i\left\{H,\right\}=-i\sum_{i=1}^{n}\left[\frac{\partial H}{\partial p_{i}}\frac{\partial }{\partial q_{i}}-\frac{\partial H}{\partial q_{i}}\frac{\partial }{\partial p_{i}}\right]$$

the Liouville operator, where $\left\{A,B\right\}=\sum_{i\;=1}^{n}\left[\frac{\partial A}{\partial p_{i}}\frac{\partial B}{\partial q_{i}}-\frac{\partial A}{\partial q_{i}}\frac{\partial B}{\partial p_{i}}\right]$ is the Poisson bracket.

Then, dynamics of the system in terms of the density $\rho_{t}$ of its states in the phase space is defined by the Liouville equation

$$i\frac{\partial \rho_{t}}{\partial t}=\left[H,\rho_{t}\right]=L\rho_{t}.$$

Prigogine and his group [10,11,29] suggested to consider the operator L as a “generator of dynamical evolution” ([10], p. 4769) of the system and defined *internal time of a dynamical system* as an operator T such that

$$i\left[L,T\right]=I.$$

where I is an identity operator.

In the quantum mechanical formalism [30], operator T of internal time is also considered a “square root” of the entropy operator M, that is

$$M=T^{H}T.$$

where $T^{H}$ stands for Hermitian conjugate of the operator T.

The external or reference time t is derived for the internal time T and is defined as an average [30]:

$$t=\;\langle T\rangle =\;\mathrm{tr}\rho_{t}^{H}T\rho_{t}.$$

Note that in this formulation, the behavior of internal time also demonstrates Markovian properties [11].

### Appendix A.3. Internal Time of Dynamical System

Denote by S be a dynamical system with the phase space X and assume that S is linear and is defined by the first-order m-dimensional differential equations [14]

$$\frac{dx}{dt}=A\left(t\right)x\left(t\right).$$

where $x\left(t\right)\in X\subset R^{m}$ are coordinates and $A\left(t\right)$ is a real m×m matrix.

An initial state $x\left(t_{0}\right)$ of the system and the values of the matrix $A\left(t\right)$ completely define the evolution of the system. If the state $x\left(t_{0}\right)$ of the system S at initial time $t_{0}$ is strictly specified, then the dynamics of the system is completely predictable and its state $x\left(t\right)$ for any $t>t_{0}$ can be calculated.

However, if $\mathrm{tr}A\left(t\right)>0$ for all t, that is, the system S is globally exploding, and the initial state $x\left(t_{0}\right)$ is unknown and is defined by a probability distribution over the state space of S, then the further states $x\left(t\right)$, $t>t_{0}$, are also defined by certain probability distributions.

Assume that the system S is globally exploding and denote by $H\left(t\right)$ the entropy of the probability distribution of system states at time t.

Then given the entropy $H\left(t_{0}\right)$, for any $t>t_{0}$, the following holds [13]:

$$H\left(t\right)=H\left(t_{0}\right)+\int_{t_{0}}^{t}\mathrm{tr}A\left(\tau \right)d\tau .$$

Since $\mathrm{tr}A\left(t\right)>0$ for all t, the entropy $H\left(t\right)$ of the system increases with t.

Using this equation, Valleé defines the value [14]

$$t\left(t\right)=H\left(t\right)-H\left(t_{0}\right)=\int_{t_{0}}^{t}\mathrm{tr}A\left(\tau \right)d\tau ,$$

which is called the internal (or intrinsic) time adapted to the dynamics of the globally exploding system or *internal time of the dynamical system* in brief.

Later [15], Valleé demonstrated that if the state $x\left(t\right)$ of the system S belongs to finite dimensional linear space, in particular, if $x\left(t\right)\in R^{m}$, then the intensity $\mathrm{tr}A\left(\tau \right)$ of the changing system at time τ can be represented by an increasing function $V:R^{+}\to R^{+}$ such that $V\left(0\right)=0$.

The simplest example of such function is the square of the Euclidian norm $V\left(\left\|\frac{dx}{dt}\right\|\right)\equiv {\left(\frac{dx}{dt}\right)}^{2}$. Then, the internal time of the dynamical system is defined as an internal duration

$$\tilde{t}\left(t\right)=d\left(t_{0},t\right)=\int_{t_{0}}^{t}{\left(\frac{dx}{d\tau }\right)}^{2}d\tau ,$$

and the duration in the interval $\left(t_{1},t_{2}\right)$ is defined as a difference $d\left(t_{1},t_{2}\right)=\tilde{t}\left(t_{2}\right)-\tilde{t}\left(t_{1}\right)$ between internal times $\tilde{t}\left(t_{2}\right)$ and $\tilde{t}\left(t_{1}\right)$ at the bounds $t_{2}$ and $t_{12}$ of the interval.

## Appendix B

In the paper, the Sinai formulation [4] of the Kolmogorov entropy [2,3] is used. For completeness, additional definitions of the related concepts are presented below.

Let X be a compact set and ε>0 be a real number.

A set $\alpha \;=\left\{A:A\subset X\right\}$ is called ε-covering of the set X if $X\subseteq \underset{A\in \alpha }{⋃}A$ and the diameter of any A∈α is not greater than 2ε.

Set X is said to be ε-distinguishable if any two of its distinct points are located at a distance greater than ε.

Denote by $N_{\varepsilon }\left(X\right)$ the minimal number of sets in ε-covering α of the set X, and by $M_{\varepsilon }\left(X\right)$ the maximal number of points in the ε-distinguishable subset of the set X.

The value [32]

$$H_{\varepsilon }\left(X\right)=\log_{2}N_{\varepsilon }\left(X\right)\;$$

is called ε-*entropy* of the set X and the value

$$E_{\varepsilon }\left(X\right)=\log_{2}M_{\varepsilon }\left(X\right)$$

is called ε-*capacity* of the set X.

These values are interpreted as follows: ε-entropy $H_{\varepsilon }\left(X\right)$ is a minimal number of bits required to transmit the set X with the precision ε, and ε-capacity $E_{\varepsilon }\left(X\right)$ is a maximal number of bits, which can be memorized by X with the precision ε.

Note that the concepts of ε-entropy and ε-capacity differ from the concept of Kolmogorov complexity, which is defined as follows.

Let σ be a sequence of symbols. Denote by $p\left(s\right)$ a program that describes the string σ and by $l\left(p\left(\sigma \right)\right)$ the length of the program $p\left(s\right)$.

The value [33]

$$K\left(s\right)=\left\{\begin{array}{c} \underset{p\left(\sigma \right)}{\min}l\left(p\left(\sigma \right)\right),\;\mathrm{if}\;p\left(\sigma \right)\;\mathrm{exists},\\ \infty ,\;\;\;\;\mathrm{if}\;p\left(\sigma \right)\;\mathrm{does}\;\mathrm{not}\;\mathrm{exist}, \end{array}\right.\;$$

where the minimum is taken over all programs describing σ, is called the *Kolmogorov complexity* of the string σ.

It can be demonstrated that Kolmogorov complexity of the trajectories of dynamical system is almost certainly equivalent to the entropy of the dynamical system $h\left(\varphi \right)$ given in Definition 1.

Now let us return to the dynamical system $S=\left(X,\varphi ,\mu \right)$. The concept that generalizes the Sinai metric entropy of dynamical system is the topological entropy defined by Agler, Konheim and McAndrew [34]. In contrast to Section 2, definition of topological entropy deals with topological space X and its coverings. Along with this, the definition follows similar steps.

Let $\beta \;=\left\{B_{1},B_{2},\ldots ,B_{n\left(\beta \right)}\right\}$, ${⋃}_{i\;=1}^{n\left(\beta \right)}B_{i}=X$, be a finite open covering of the space X. Then, the set

$$\varphi^{\nu }\left(\beta \right)=\left\{\varphi^{\nu }\left(B_{1}\right),\varphi^{\nu }\left(B_{2}\right),\ldots ,\varphi^{\nu }\left(B_{n\left(\alpha \right)}\right)\right\},\;\nu =0,\;1,\;2,\ldots ,$$

resulting from the νth application of the automorphism φ to partition β is also an open covering of X.

Let $N\left(\varphi^{N}\right)$ be cardinality of the minimal subcovering of the covering

$$\beta_{N}\left(\varphi \right)={⋁}_{v=0}^{N}\varphi^{v}\left(\beta \right).$$

The value [34]

$$h\left(\varphi ,\beta \right)=\underset{N\to \infty }{\lim}\frac{1}{N}\log N\left(\varphi^{N}\right),$$

is called the *topological entropy of the system* S *with respect to time*, and its supremum

$$h\left(\varphi \right)=\sup_{\beta }h\left(\varphi ,\beta \right),$$

taken over all open covering of X is called the *topological entropy* of dynamical system S.

Dinaburg demonstrated [35] the following relations between topological entropy $h\left(\varphi \right)$, metric entropy $h_{\mu }\left(\varphi \right)$, and ε-entropy $H_{\varepsilon }\left(X\right)$, namely, the following equalities hold

$$h\left(\varphi \right)=\underset{\varepsilon \to 0}{\lim}\underset{v\to \infty }{\lim}H_{\varepsilon }\left(X\right)\;\;\;\mathrm{and}\;\;\;h\left(\varphi \right)=\sup_{\beta }h_{\mu }\left(\varphi \right),$$

where the supremum is taken over all invariants with respect to φ measures on X.

Finally, since at each iteration, automorphism φ acts on a finite set of subsets of X, internal time of the system can be considered in terms of symbolic dynamics and algebraic entropy suggested by Goppa [36] in the framework of coding theory.

Let U be a finite set of symbols (alphabet) of the size $n\left(U\right)$ and $\Omega =U^{n}$ be a set of all words of length n in alphabet U. Denote by G a symmetric group that permutes the letters of the words such that if $\omega =\left(u_{1},\;u_{2},\ldots ,u_{n}\right)\in \Omega$, then

$$g\left(\omega \right)=\left(u_{g\left(1\right)},\;u_{g\left(2\right)},\ldots ,u_{g\left(n\right)}\right)\in \Omega ,\;g\in G.$$

The value [36]

$$I_{0}\left(\omega \right)=\log\left|G\left(\omega \right)\right|=\frac{n!}{m_{1}!m_{2}!\ldots m_{n\left(U\right)}!},$$

where $m_{i}$, $i=1,2,\ldots ,n\left(U\right)$, is the number of times that the letter $u_{i}\in U$ appears in the word ω, is called *null-information* or *algebraic entropy* of the word ω.

## Appendix C

For illustration, detailed calculations for Examples 2 and 3 are presented below.

### Appendix C.1. Calculations for Example 2

Recall that everywhere, log is taken base 2. We have

$$\varphi^{0}\left(\alpha ,2\right)=\left\{\left(\left.0,\frac{1}{2}\right]\right.,\;\left(\left.\frac{1}{2},1\right]\right.\right\},\;\mu_{1}=\mu \left(\left.0,\frac{1}{2}\right]\right.=\frac{1}{2}-0=\frac{1}{2},\;\mu_{2}=\mu \left(\left.\frac{1}{2},1\right]\right.=1-\frac{1}{2}=\frac{1}{2},\;H\left[\varphi^{0}\left(\alpha ,2\right)\right]=-\mu_{1}\log\mu_{1}-\mu_{2}\log\mu_{2}=\;=-\frac{1}{2}\log\frac{1}{2}-\frac{1}{2}\log\frac{1}{2}=-2\left(\frac{1}{2}\log\frac{1}{2}\right)=-\log\frac{1}{2}=1;\;\varphi^{1}\left(\alpha ,2\right)=\left\{\left(\left.0,\frac{1}{4}\right]\right.,\;\left(\left.\frac{1}{4},\frac{1}{2}\right]\right.,\left(\left.\frac{1}{2},\frac{3}{4}\right]\right.,\;\left(\left.\frac{3}{4},1\right]\right.\right\},\;\mu_{1}=\mu \left(\left.0,\frac{1}{4}\right]\right.=\frac{1}{4}-0=\frac{1}{4},\;\mu_{2}=\mu \left(\left.\frac{1}{4},\frac{1}{2}\right]\right.=\frac{1}{2}-\frac{1}{4}=\frac{1}{4},\;\mu_{3}=\mu \left(\left.\frac{1}{2},\frac{3}{4}\right]\right.=\frac{3}{4}-\frac{1}{2}=\frac{1}{4},\;\mu_{4}=\mu \left(\left.\frac{3}{4},1\right]\right.=1-\frac{3}{4}=\frac{1}{4},\;H\left[\varphi^{1}\left(\alpha ,2\right)\right]=-\mu_{1}\log\mu_{1}-\mu_{2}\log\mu_{2}-\mu_{3}\log\mu_{3}-\mu_{4}\log\mu_{4}=\;=-\frac{1}{4}\log\frac{1}{4}-\frac{1}{4}\log\frac{1}{4}-\frac{1}{4}\log\frac{1}{4}-\frac{1}{4}\log\frac{1}{4}=\;-4\left(\frac{1}{4}\log\frac{1}{4}\right)-\log\frac{1}{4}=2;\;\varphi^{2}\left(\alpha ,2\right)=\left\{\left(\left.0,\frac{1}{8}\right]\right.,\;\left(\left.\frac{1}{8},\frac{1}{4}\right]\right.,\left(\left.\frac{1}{4},\frac{3}{8}\right]\right.,\;\left(\left.\frac{3}{8},\frac{1}{2}\right]\right.,\left(\left.\frac{1}{2},\frac{5}{8}\right]\right.,\;\left(\left.\frac{5}{8},\frac{3}{4}\right]\right.,\left(\left.\frac{3}{4},\frac{7}{8}\right]\right.,\;\left(\left.\frac{7}{8},1\right]\right.\right\},\;\mu_{1}=\mu \left(\left.0,\frac{1}{8}\right]\right.=\frac{1}{8}-0=\frac{1}{8},\;\mu_{2}=\mu \left(\left.\frac{1}{8},\frac{1}{4}\right]\right.=\frac{1}{4}-\frac{1}{8}=\frac{1}{8}\;\;\ldots \;\ldots ,\;\mu_{7}=\mu \left(\left.\frac{3}{4},\frac{7}{8}\right]\right.=\;\frac{7}{8}-\frac{3}{4}=\frac{1}{8},\;\mu_{8}=\mu \left(\left.\frac{7}{8},1\right]\right.=1-\frac{7}{8}=\frac{1}{8},\;H\left[\varphi^{2}\left(\alpha ,2\right)\right]==-\mu_{1}\log\mu_{1}-\mu_{2}\log\mu_{2}-\ldots -\mu_{7}\log\mu_{7}-\mu_{8}\log\mu_{8}=\;=\underset{8\;times}{\underbrace{-\frac{1}{8}\log\frac{1}{8}-\frac{1}{8}\log\frac{1}{8}-\ldots -\frac{1}{8}\log\frac{1}{8}-\frac{1}{8}\log\frac{1}{8}}}=\;=-8\left(\frac{1}{8}\log\frac{1}{8}\right)=\log\frac{1}{8}=3.$$

Then,

$$\tau_{1}\left(\varphi \right)=\frac{1}{H\left[\varphi^{0}\left(\alpha \right)\right]}\left(H\left[\varphi^{1}\left(\alpha ,2\right)\right]-H\left[\varphi^{0}\left(\alpha ,2\right)\right]\right)=\frac{1}{1}\left(2-1\right)=1,\;\tau_{2}\left(\varphi \right)=\frac{1}{H\left[\varphi^{0}\left(\alpha \right)\right]}\left(H\left[\varphi^{2}\left(\alpha ,2\right)\right]-H\left[\varphi^{1}\left(\alpha ,2\right)\right]\right)=\frac{1}{1}\left(3-2\right)=1,$$

and

$$\tau \left(\varphi \right)=1.$$

### Appendix C.2. Calculations for Example 3

Let

$$\varphi^{0}\left(\alpha \right)=\left\{\left[\left.0,\frac{1}{2}\right)\right.,\;\left[\frac{1}{2},1\right]\right\},\;\varphi^{1}\left(\alpha \right)=\left\{\left[\left.0,\frac{1}{2}\right)\right.,\;\left[\frac{1}{2},1\right]\right\}\mathrm{and}\;\varphi^{2}\left(\alpha \right)=\left\{\left[\left.0,\frac{1}{2}\right)\right.,\;\left[\frac{1}{2},1\right]\right\}.$$

The entropies of these partitions are equivalent:

$$H\left[\varphi^{0}\left(\alpha \right)\right]=\;H\left[\varphi^{1}\left(\alpha \right)\right]=\;H\left[\varphi^{2}\left(\alpha \right)\right].$$

Similar to above, for these partitions, we have

$$\mu_{1}=\mu \left(\left.0,\frac{1}{2}\right]\right.=\frac{1}{2}-0=\frac{1}{2},\;\mu_{2}=\mu \left(\left.\frac{1}{2},1\right]\right.=1-\frac{1}{2}=\frac{1}{2},$$

and

$$H\left[\varphi^{0}\left(\alpha ,2\right)\right]=H\left[\varphi^{1}\left(\alpha \right)\right]=H\left[\varphi^{2}\left(\alpha \right)\right]=\;=-\mu_{1}\log\mu_{1}-\mu_{2}\log\mu_{2}=\;=-\frac{1}{2}\log\frac{1}{2}-\frac{1}{2}\log\frac{1}{2}=-2\left(\frac{1}{2}\log\frac{1}{2}\right)=-\log\frac{1}{2}=1;$$

Then,

$$\tau_{1}\left(\varphi \right)=\frac{1}{H\left[\varphi^{0}\left(\alpha \right)\right]}\left(H\left[\varphi^{1}\left(\alpha ,2\right)\right]-H\left[\varphi^{0}\left(\alpha ,2\right)\right]\right)=\frac{1}{1}\left(1\;-1\right)=0,\;\tau_{2}\left(\varphi \right)=\frac{1}{H\left[\varphi^{0}\left(\alpha \right)\right]}\left(H\left[\varphi^{2}\left(\alpha ,2\right)\right]-H\left[\varphi^{1}\left(\alpha ,2\right)\right]\right)=\frac{1}{1}\left(1-1\right)=0,$$

and

$$\tau \left(\varphi \right)=0.$$

Now, let

$$\varphi^{0}\left(\alpha \right)=\left\{\left[\left.0,\frac{1}{3}\right)\right.,\;\left[\frac{1}{3},1\right]\right\}.$$

Then,

$$\mu_{1}=\mu \left(\left.0,\frac{1}{3}\right]\right.=\frac{1}{3}-0=\frac{1}{3},\;\mu_{2}=\mu \left(\left.\frac{1}{3},1\right]\right.=1-\frac{1}{3}=\frac{2}{3},\;H\left[\varphi^{0}\left(\alpha \right)\right]=-\mu_{1}\log\mu_{1}-\mu_{2}\log\mu_{2}=\;=-\frac{1}{3}\log\frac{1}{3}-\frac{2}{3}\log\frac{2}{3}=\log3-\frac{2}{3}.$$

Similarly, for

$$\varphi^{1}\left(\alpha \right)=\left\{\left[\left.0,\frac{2}{3}\right)\right.,\;\left[\frac{2}{3},1\right]\right\}$$

we have

$$\mu_{1}=\mu \left(\left.0,\frac{2}{3}\right]\right.=\;\frac{2}{3}-0=\frac{2}{3},\;\mu_{2}=\mu \left(\left.\frac{2}{3},1\right]\right.=1-\frac{2}{3}=\frac{1}{3},\;H\left[\varphi^{1}\left(\alpha \right)\right]=-\mu_{1}\log\mu_{1}-\mu_{2}\log\mu_{2}=\;=-\frac{2}{3}\log\frac{2}{3}-\frac{1}{3}\log\frac{1}{3}=\log3-\frac{2}{3}.$$

and for

$$\varphi^{2}\left(\alpha \right)=\left\{\left[\left.0,\frac{1}{3}\right)\right.,\;\left[\frac{1}{3},1\right]\right\}$$

we have

$$\mu_{1}=\mu \left(\left.0,\frac{1}{3}\right]\right.=\frac{1}{3}-0=\frac{1}{3},\;\mu_{2}=\mu \left(\left.\frac{1}{3},1\right]\right.=1-\frac{1}{3}=\frac{2}{3},\;H\left[\varphi^{2}\left(\alpha \right)\right]=-\mu_{1}\log\mu_{1}-\mu_{2}\log\mu_{2}=\;=-\frac{1}{3}\log\frac{1}{3}-\frac{2}{3}\log\frac{2}{3}=\log3-\frac{2}{3}.$$

Finally, for

$$\varphi^{1}\left(\alpha \right)\vee \varphi^{0}\left(\alpha \right)=\varphi^{2}\left(\alpha \right)\vee \varphi^{1}\left(\alpha \right)=\left\{\left[\left.0,\frac{1}{3}\right)\right.,\left[\left.\frac{1}{3},\frac{2}{3}\right)\right.,\;\left[\frac{2}{3},1\right]\right\}$$

we have

$$\mu_{1}=\mu \left[\left.0,\frac{1}{3}\right)\right.=\frac{1}{3}-0=\frac{1}{3},\;\mu_{2}=\mu \left[\left.\frac{1}{3},\frac{2}{3}\right)\right.=\frac{2}{3}-\frac{1}{3}=\frac{1}{3}\;\mathrm{and}\;\mu_{3}=\mu \left[\frac{2}{3},1\right]=1-\frac{2}{3}=\frac{1}{3},\;H\left[\varphi^{1}\left(\alpha \right)\vee \varphi^{0}\left(\alpha \right)\right]=H\left[\varphi^{2}\left(\alpha \right)\vee \varphi^{1}\left(\alpha \right)\right]=-\mu_{1}\log\mu_{1}-\mu_{2}\log\mu_{2}-\mu_{3}\log\mu_{3}=\;=-\frac{1}{3}\log\frac{1}{3}-\frac{1}{3}\log\frac{1}{3}-\frac{1}{3}\log\frac{1}{3}=\log3.$$

Then,

$$\tau_{1}\left(\varphi \right)=\frac{1}{H\left[\varphi^{0}\left(\alpha \right)\right]}\left(H\left[\varphi^{1}\left(\alpha \right)\vee \varphi^{0}\left(\alpha \right)\right]-H\left[\varphi^{0}\left(\alpha \right)\right]\right)=\;=\frac{1}{\log3-\frac{2}{3}}\left(\log3-\left(\log3-\frac{2}{3}\right)\right)=\frac{2}{3\left(\log3-\frac{2}{3}\right)}=\frac{2}{3\log3-2}\approx 0.726,$$

$$\tau_{2}\left(\varphi \right)=\frac{1}{H\left[\varphi^{0}\left(\alpha \right)\right]}\left(H\left[\varphi^{2}\left(\alpha \right)\vee \varphi^{1}\left(\alpha \right)\right]-H\left[\varphi^{1}\left(\alpha \right)\right]\right)=\;=\frac{1}{\log3-\frac{2}{3}}\left(\log3-\left(\log3-\frac{2}{3}\right)\right)=\frac{2}{3\log3-2}\approx 0.726,$$

and

$$\tau \left(\varphi \right)=\frac{2}{3\log3-2}\approx 0.726.$$
